# Complete Genome Sequence of Micrococcus yunnanensis TT9, Isolated from a Healthy Volunteer

**DOI:** 10.1128/mra.00652-22

**Published:** 2022-08-15

**Authors:** Yujin Choi, Haelim Son, Kyoung Lee

**Affiliations:** a Department of Bio Health Science, Changwon National University, Changwon, Republic of Korea; University of Rochester School of Medicine and Dentistry

## Abstract

Micrococcus yunnanensis TT9 was isolated from the forehead of human skin. This strain can grow on Triton X-100. We report the complete whole-genome sequence of this strain, which has one chromosome of 2,470,932 bp (73.0% G+C content) with 2,151 coding sequences.

## ANNOUNCEMENT

Micrococcus yunnanensis is a high-GC-content, Gram-positive coccus phylogenetically belonging to the phylum *Actinobacteria*. Its involvement in the degradation of pollutants such as polycyclic aromatic hydrocarbons on the human skin has been reported (1). This article reports the isolation and whole-genome sequence of *M. yunnanensis* TT9 from human skin, which can grow on Triton X-100.

A swab sample from the skin of a male college student’s forehead was directly streaked on minimal salts basal medium (MSB) agar (2) containing 0.02% yeast extract and 0.5% Triton X-100 (Sigma-Aldrich). The agar medium was aerobically incubated for 1 week at 28°C for growth. One strain, named TT9, was further purified by streaking on tryptic soy broth (TSB) agar (Kisan) and incubation at 28°C for 2 days. This strain forming light-yellow colored colonies was deposited in the Korean Collection for Type Cultures as KCTC 49797. Ethical approval for subject sampling was granted by the institutional review board of Changwon National University. A similarity search for sequences in the type material of the NCBI database produced the best hits with the 16S rRNA gene sequences of Micrococcus luteus NCTC 2665 (accession number NR_075062.2), *M. aloeverae* AE-6 (NR_134088.1), and *M. yunnanensis* YIM 65004 (NR_116578.1), with more than 99% identities.

For DNA extraction, cells were cultured in a flask in TSB for 48 h at 28°C with shaking at 140 rpm. Total genomic DNA was purified using the phenol extraction method (3). Genomic DNA was sequenced with Illumina and Oxford Nanopore Technologies. Illumina sequencing was performed at DNALink Co. (Seoul, Korea). The whole-genome sequencing was performed using a TruSeq DNA PCR-free 550 bp library kit (Illumina) and demultiplexing by bcl2fastaq2 (ver. 2.20) on the Illumina NovaSeq6000 sequencer with default settings. The quality of the raw sequencing data was checked using FastQC with ASCII Qscore offset 33 (https://www.bioinformatics.babraham.ac.uk/projects/fastqc/) (4). The read length was 2 × 151 of approximately 550 bp insert size. A total of 40,082,478 reads with 6,052 Mbp with a mean Phred quality score of 35.52 were produced. According to the instructions, libraries for Nanopore sequencing were prepared with the SQK-LSK109 kit and multiplexed using the EXP-NBD104 barcoding kit. Sequencing was performed on a MinION sequencer (v20.10.3) using an R9.4.1 flow cell with default settings. Reads were base called and demultiplexed using Guppy v3.4.1 in high accuracy mode with the minimum Qscore of 8. The final data set (reads, 8,756; total base number, 140.36 Mbp; mean length, 16,029 bp; minimum, 112 bp; maximum, 161,826 bp; *N*_50_, 30,795 bp) was yielded and was assembled *de novo* following a hybrid Nanopore–Illumina program using Unicycler v0.4.9 (https://github.com/rrwick/Unicycler) (5) with default settings. The quality of the assembled genome sequences was evaluated using CheckM v1.1.3 (https://github.com/Ecogenomics/CheckM/releases/tag/v1.1.3) (6). Gene predictions and annotations were provided by NCBI using the best-placed reference protein set and GeneMarkS-2+ of the NCBI Prokaryotic Genome Annotation Pipeline 6.1 (7). In addition, the subsystem features in the genome were analyzed by Rapid Annotations using Subsystems Technology (RAST) (https://rast.nmpdr.org/) (8). The relationship of the BLAST average nucleotide identity (ANIb) values of strain TT9 and type species in the database was searched in the JSpeciesWS server (https://jspecies.ribohost.com/jspeciesws/) (9). Digital DNA–DNA hybridization (dDDH) values were calculated by applying the Genome-to-Genome Distance calculator (GGDC 3.0) using formula 2 (https://ggdc.dsmz.de/ggdc.php) (10).

The final genome assembly of TT9 was found to be closed, circular, and rotated to place *dnaA* at the origin of replication and consists of one chromosome of 2,470,932 bp (73.0% G+C content), with 2,507 × mean coverage. The plasmid was not detected. Analysis with CheckM showed 98.70% completeness and 0% strain heterogeneity. TT9 exhibited the highest similarity with *M. yunnanensis* DSM 21948^T^ with an ANI value of 98.17% (84.0% coverage) and dDDH of 85.5% (formula 2).

**FIG 1:**
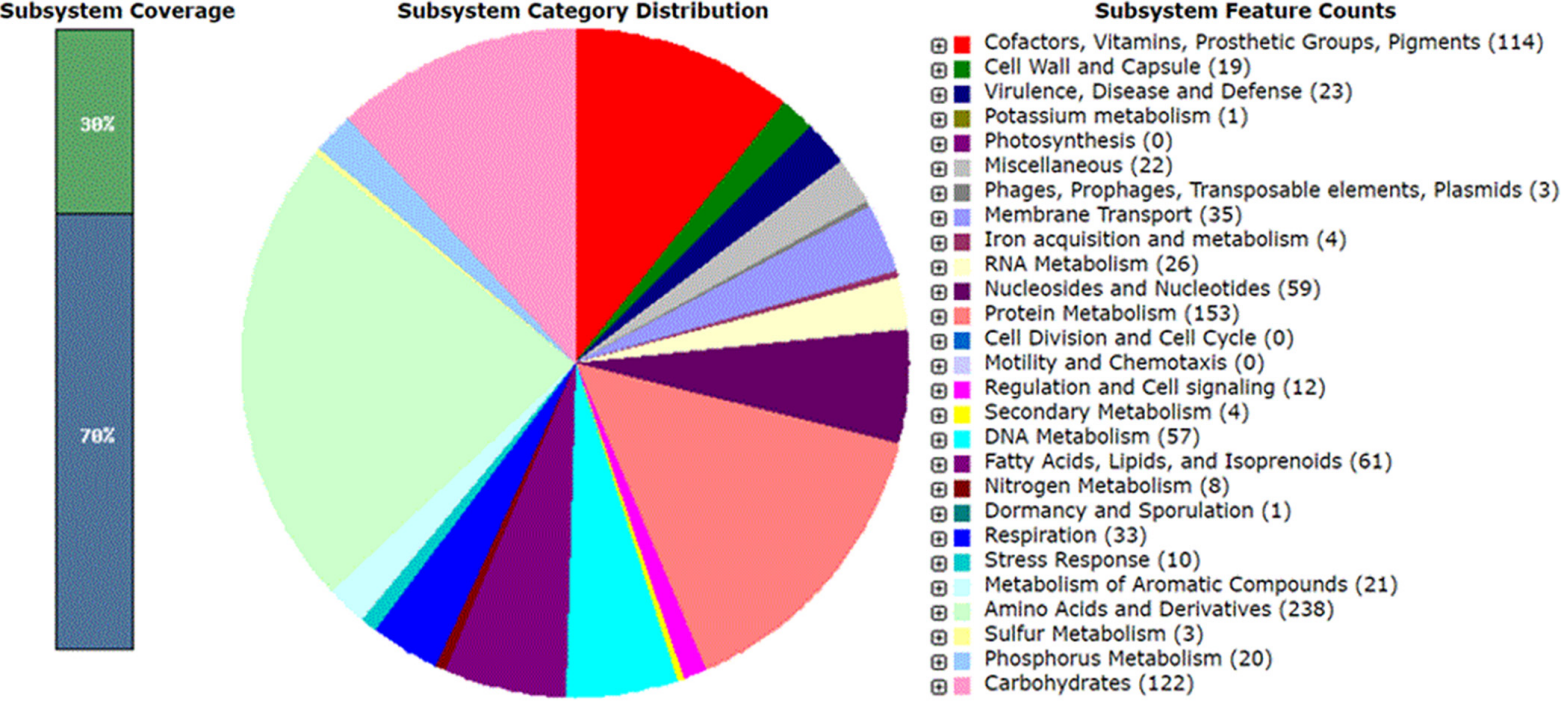
Functional category distribution assigned to the genome of *M. yunnanensis* TT9 according to RAST analysis.

The chromosome contains 2,151 protein-coding sequences, two copies of rRNA genes (5S, 16S, and 23S), 48 coding regions of tRNAs, and three ncRNA genes. An overview of the subsystem features (functional category distribution) assigned to the genome of *M. yunnanensis* TT9 is shown in Fig. 1. A total of 237 subsystems (30% of coding genes) were identified. The *M. yunnanensis* TT9 genome contained genes coding for the glyoxylate cycle required for Triton X-100 degradation by Pseudomonas nitroreducens TX1 (11).

### Data availability.

The *M. yunnanensis* TT9 genome sequence has been deposited in GenBank under accession number CP097650.1. Raw sequence data used for assembly were deposited in GenBank under SRA accession numbers SRX15389513 (MinIon seq) and SRX15389512 (Illumina Nova seq).
